# Evaluation of A Concentrated Preterm Formula as a Liquid Human Milk Fortifier in Preterm Babies at Increased Risk of Feed Intolerance

**DOI:** 10.3390/nu10101433

**Published:** 2018-10-04

**Authors:** Anish Pillai, Susan Albersheim, Julie Matheson, Vikki Lalari, Sylvia Wei, Sheila M Innis, Rajavel Elango

**Affiliations:** 1 Neonatal-Perinatal Medicine, British Columbia Women’s and Children’s Hospital, University of British Columbia, Vancouver, BC V6H 3N1, Canada; anish.pillai@cw.bc.ca (A.P.); salbersheim@cw.bc.ca (S.A.); vlalari@cw.bc.ca (V.L.); 2 Department of Pediatrics, University of British Columbia, Vancouver, BC V5Z 3V4 Canada; Sinnis@bcchr.ubc.ca; 3 British Columbia Children’s Hospital Research Institute (BCCHRI), Vancouver, BC V5Z 4H4 Canada; jmatheson@bcchr.ca (J.M.); sylviaawei@gmail.com (S.W.); 4 School of Population and Public Health, University of British Columbia, Vancouver, BC V6T 1Z3 Canada

**Keywords:** premature, formula, fortification, human milk

## Abstract

There are concerns around safety and tolerance of powder human milk fortifiers to optimize nutrition in preterm infants. The purpose of this study was to evaluate the tolerance and safety of a concentrated preterm formula (CPF) as a liquid human milk fortifier (HMF) for premature infants at increased risk of feeding intolerance. We prospectively enrolled preterm infants over an 18-month period, for whom a clinical decision had been made to add CPF to human milk due to concerns regarding tolerance of powder HMF. Data on feed tolerance, anthropometry, and serum biochemistry values were recorded. Serious adverse events, such as mortality, necrotizing enterocolitis (NEC), and sepsis, were monitored. A total of 29 babies received CPF fortified milk during the study period. The most common indication for starting CPF was previous intolerance to powder HMF. Feeding intolerance was noted in 4 infants on CPF. The growth velocity of infants was satisfactory (15.9 g/kg/day) after addition of CPF to feeds. The use of CPF as a fortifier in preterm babies considered at increased risk for feed intolerance seems well tolerated and facilitates adequate growth. Under close nutrition monitoring, this provides an additional option for human milk fortification in this challenging subgroup of preterm babies, especially in settings with limited human milk fortifier options.

## 1. Introduction

Human milk is considered the ideal food for neonates and is the preferred choice of diet for preterm and very low birth weight infants [1,2,3]. The benefits of human milk for preterm infants include a lower risk of necrotizing enterocolitis (NEC) and severe infections, improved feed tolerance, improved cognition and development, and shorter hospital stay [4,5,6,7]. These benefits are probably related to the multiple growth factors, immunological constituents, and bioactive factors in human milk that are absent from infant formula. However, preterm breast milk is deficient in certain nutrients and does not meet the nutritional requirements of a premature baby [2,8,9]. Feeding premature babies with unfortified human milk is associated with poor growth rate, reduced bone mineral density, and poorer short-term developmental outcome [10,11,12].

Human milk fortifier (HMF) is an additive designed to boost the total energy, protein, and micronutrient content of human milk to enhance growth in preterm infants [13]. The key benefits of fortification are improved short-term growth, bone mineralization, and protein status. However, there have been concerns regarding an increased risk of feed intolerance and NEC in preterm babies on HMF [14,15,16,17,18]. Additives can increase the osmolality of milk, which has been associated with mucosal injury and reduced gut motility [19,20,21,22]. It thus becomes challenging to add nutritional supplements to feeds in a baby with a history of intolerance to HMF or prior NEC and achieve adequate growth.

Human milk fortifiers are available in different compositions, specifically varying in protein (source and amounts), micronutrient composition, and form (powder or liquid). The use of a liquid product might be preferred over powder in the Neonatal Intensive Care Unit (NICU) setting to reduce risk of contamination and infection [23]. Our NICU routinely uses a commercially available powder HMF preparation; however, there have been concerns among clinicians regarding the tolerance of a powdered fortifier in premature babies with a previous history of NEC, surgical bowel, or feed intolerance. In our NICU, preterm infants who are postsurgical for intestinal problems are considered at increased risk for development of intestinal calculi (milk curd syndrome) when powder HMF is added to their feeds [24]. The intestinal concretions of powder HMF may be significant enough to cause acute deterioration and warrant surgical intervention due to intestinal obstruction [24,25,26]. Unlike powdered products, liquid HMF has the advantage of sterility and easier mixing with human milk. Liquid fortifiers also provide an additional option for feeding preterm infants whose mothers have insufficient milk supply to fulfil the infant’s needs, since the use of a liquid fortifier both extends the volume of milk and decreases the need for using powder HMF.

The use of powder HMF is standard practice in our NICU and liquid HMF is not available for use in preterm babies. Health Canada approved Similac Special Care 30 (SSC30) concentrated preterm formula (CPF) for use in premature babies in NICUs in Canada in early 2014. CPF can be used as a high calorie standalone feed or mixed with less concentrated preterm formulas to achieve desired caloric density. Although there is limited literature on the use of SSC30 (CPF) as a liquid human milk fortifier in the NICU [27], there is no experience or data in the subgroup of preterm babies at increased risk for feed intolerance. At the time of this study, there were no other liquid human milk fortifier products available in Canada. The primary objective of this project was to assess the post-marketing safety and tolerance of CPF as a liquid human milk fortifier in a special group of premature babies considered at increased risk for feed intolerance. The secondary objectives were to describe the short-term outcomes, including growth, serum chemistries, and morbidities with CPF use. This cohort comprised mainly babies with previous intolerance to powder HMF or babies with intestinal perforation/NEC, for whom the clinical team did not feel comfortable in adding or restarting powder HMF.

## 2. Materials and Methods

Study population/setting: The study was conducted in a 60 bed tertiary care NICU at BC Women’s Hospital and Health Centre, in Vancouver, British Columbia. The unit cares for approximately 670 babies per year, admitted from across British Columbia and the Yukon Territory. Babies for whom a clinical decision had been made to add CPF as a liquid nutrient fortifier to mother’s own milk (MOM) or pasteurized donor human milk (DHM) provided by BC Women’s Provincial Milk Bank, located within the hospital, were eligible for the study. All babies who received CPF during the 18-month enrolment period were included. The clinical decision to start CPF was made if there was significant intolerance after adding powder HMF or if the clinical team felt the baby would not be a good candidate for powder products. The potential benefit of CPF in this cohort could be due to its lower osmolality, easier solubility, and non-acidified nature. However, it must be noted that the powder HMF used in our cohort was also non-acidified, and the small difference in osmolality may not have clinical significance. The composition of CPF in comparison to powder HMF is provided in Table 1.

Exclusion criteria were infants with congenital/chromosomal anomalies, and multiorgan or intestinal dysfunction that in the opinion of the infant’s primary physician was not compatible with survival or attainment of full enteral feeds. Enteral feeding was initiated and advanced as per existing unit policy with the use of standardized powder HMF protocol initiated at 22 kcal/oz or 74 kcal/100 mL (2 packets per 100 mL), and advanced to a target calorie content of 24 kcal/oz or 81 kcal/100 mL (4 packets per 100 mL). If a decision was made to start CPF for any baby, fortification would be initiated at 10% of CPF, mixed with 90% of human milk (1:9 ratio giving 21 kcal/oz) and increased gradually by 10% each day, as tolerated. The typical target fortification would be 40% CPF with 60% human milk, to achieve a calorie content of 24 kcal/oz. However, if poor growth was noted, there was an option of increasing fortification to 25 kcal/oz with 50% CPF and 50% human milk, as per manufacturer recommendations. The babies were closely monitored by the dietitian and clinical team for nutritional adequacy.

Study design: This was a prospective observational cohort study conducted over an 18-month period from September 2014 to February 2016. Data recording for this study was performed in a predetermined, consistent format; data were extracted prospectively and confirmed retrospectively from the infants’ health records, and entered into an electronic database. Research approval was obtained from the University of British Columbia / Children’s and Women’s Health Centre of British Columbia Research Ethics Board (# H14-01452). The study did not involve changes to clinical practice or collection of additional blood samples. Since the addition of CPF displaces human milk from feeds, it was not considered ethically appropriate to do a randomized trial or have a control group. Hence, in this observational study, we only included babies for whom a clinical decision to add CPF was made by the medical team.

The primary outcome variable was feed intolerance, which was defined as feeds being withheld for 24 h or more due to concerns related to feeding. This included clinical features like abdominal distension, emesis, and change in stools/stoma output. Other outcome variables recorded were growth velocity (GV), presence of major morbidities such as NEC, late onset neonatal sepsis (LONS), chronic lung disease (CLD), retinopathy of prematurity (ROP), metabolic bone disease (MBD), and abnormal serum chemistry.

Blood was drawn initially as ordered by the clinical team for specific biochemistries or hematology until the infant was stable and growing with enteral feeds. Subsequently, biweekly samples were analysed for hematocrit, ionized calcium, alkaline phosphatase, phosphorus, albumin, prealbumin, blood urea nitrogen (BUN), sodium, and potassium. Blood gas was drawn as clinically indicated. Additional information collected included data regarding total fluid intake, parenteral nutrition and enteral feeds.

Infants were weighed daily and head circumference (HC) and length (cm) were recorded weekly, as per unit policy. Fenton growth curve percentile rankings, with Z scores, were plotted for all anthropometric measurements, enabling us to account for differences in gestational age using the revised 2013 Fenton growth charts [31,32,33]. Growth velocity (GV) was calculated using the exponential model for each infant as GV = [1000 × ln(*W*n/*W*1)]/(*D*n − *D*_1_) where W is the weight and D is the day. This exponential model has been shown to be more consistent and is validated for use in very low birth weight (VLBW) infants [34,35]. Length was measured as centimeters (cm) and length gain was calculated as cm/day. Similar calculations were used for growth in head circumference. Growth was calculated for the total NICU stay as well as specifically for the period the babies were on CPF fortified milk.

Data for major morbidities including NEC, LONS, CLD, ROP, and MBD, were collected each week from the medical charts for each infant receiving CPF. Confirmed NEC was defined as ≥Stage 2 by the Modified Bell’s staging criteria [36]. Infants who required therapy with oxygen >21% for at least 28 days were classified as having CLD at 36 weeks postmenstrual age using National Institutes of Health (NIH) consensus definition. [37]. Diagnosis of ROP and staging were categorized according to international classification for ROP or need for intervention (laser, Avastin therapy, or surgery) [38]. Babies with alkaline phosphatase levels greater than 500 IU/L were diagnosed as MBD as per our unit policy [39]

## 3. Results

**Figure 1:**
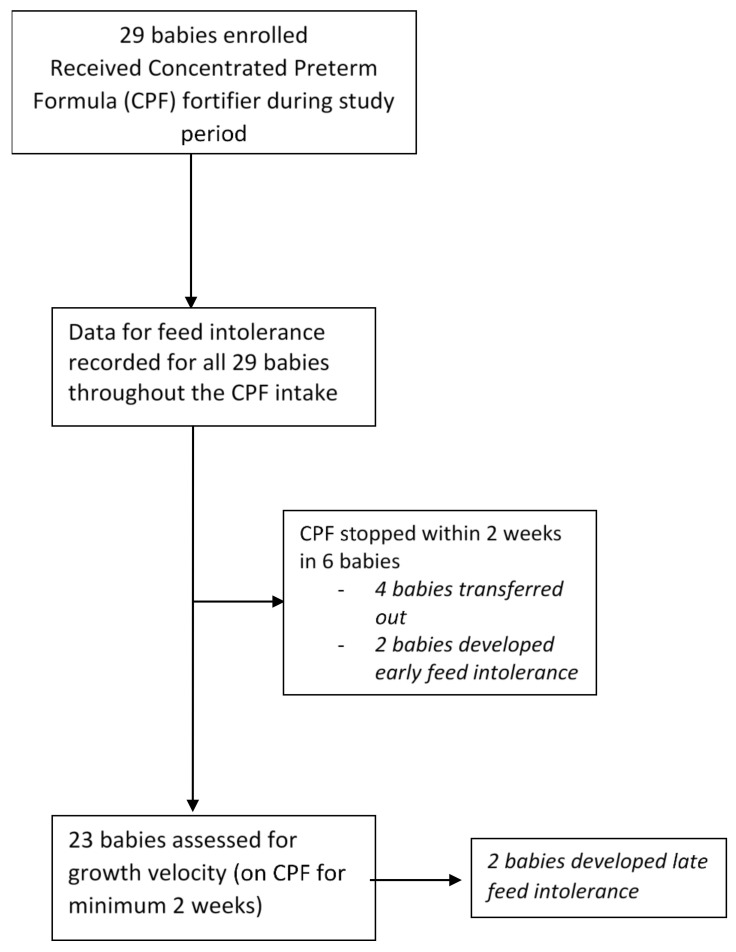
Flowchart of study participants.

Study population: A total of 29 patients received SSC 30 as fortifier during the study period. (Figure 1) Data from all infants were used to analyse the fortifier tolerance; however, only data from 23 babies who received SSC 30 fortifier for a minimum of 14 days were utilized for growth assessment. Due to the limited sample size, the data are described as median and interquartile ranges (IQR).

Patient Characteristics: The baseline characteristics and morbidities of the study population are described below (Table 2). The median day of life for achieving full feeds was 30 days, which suggests that the study cohort comprised ‘slow feeders’ due to intestinal problems or evidence of feed intolerance. The addition of CPF was done at a median age of 47 days, as many babies were initially started on powder HMF. The total duration on CPF supplementation was variable.

Indication to start CPF: Addition of CPF as a fortifier to feeds was done only for cases where the clinical team did not feel comfortable using powder HMF. Since the use of powder HMF was standard practice in our NICU, the most common indication for use of CPF fortifier was intolerance to powder HMF (*n* = 12, 41%). Indicators of intolerance to powder HMF included nil per oral status (NPO) for at least 24 h due to concerns related to feeding. The use of CPF due to surgical bowel was identified in 7 babies (24%), with NEC being the reason in 5 cases, and spontaneous intestinal perforation in 2 infants. The overall incidence of surgical NEC in our cohort was 17% (*n* = 5); 3 cases reported after powder HMF, none after CPF. This was much higher than the overall NEC incidence rate in our unit of 1.8% for the year 2015. Of all surgical babies, 3 were managed with a Penrose drain and 4 required a stoma. None of the neonates met the criteria for short bowel syndrome. In 8 babies (28%), CPF was started as the initial fortifier of choice due to concerns regarding potential tolerance of powder HMF by the medical team. This included babies who were slow to reach full feeds and showed signs of intolerance during various stages of feed advancement. In two babies (twins), the decision to add CPF was made with the dual intention of enhancement of volume for low breast milk production and a history of intolerance to powder HMF.

Tolerance: The overall incidence of feed intolerance (NPO for greater than 24 h) in the study cohort was 14% (*n* = 4). (Table 3) In one baby, the intolerance symptoms after starting CPF were thought to be unrelated to the CPF fortifier itself, and CPF was restarted after a few days with feeds. There was one case of culture positive sepsis (late onset sepsis) after being on CPF for 28 days. Two babies had ventilator associated pneumonia (clinical sepsis), requiring antibiotic therapy. None of the babies had NEC (clinical or radiologic) after addition of CPF.

Growth: The study cohort received CPF for a variable period during their NICU stay. The median number of days required to reach from start of CPF to the recommended concentration of 24 kcal/oz was 7 (5–9.5). After the addition of CPF fortifier at 24 kcal/oz, the GV of neonates (calculated by exponential model) improved from median 12.5 g/kg/day to median 15.9 g/kg/day. Median weight gain calculated as grams/day during this period on CPF was also within acceptable range (31.4 g/day). Of the 7 surgical babies, 4 babies had bowel resection and stoma placement and were followed by the intestinal rehabilitation team. The weight gain on CPF for this cohort was 18.5 g/day. Some babies (*n* = 10) received additional protein or lipid additives along with CPF, as decided by the medical team. However, the GV was satisfactory (16.8 g/kg/day) even for the cohort receiving CPF as the only fortifier and no additional supplements (*n* = 13). The overall growth in head circumference and length remained satisfactory with the addition of CPF fortifier. (Table 4)

Chemistry: One baby had elevated phosphate (2.54 mmol/L) and 2 babies had hyponatremia (both 128 mmol/L) on CPF. No abnormalities of ionized calcium or potassium were noted. The median value for urea increased from 0.9 to 2.7 mmol/L and the median pre-albumin levels improved from 66 to 103 mg/L on CPF fortified feeds. None of the neonates had alkaline phosphatase levels greater than 500 IU/L after starting CPF, and the median alkaline phosphatase values showed a downward trend from a median of 363 IU/L at the start of CPF to 245 IU/L at the end of CPF. No infant had metabolic acidosis while on CPF feeds.

## 4. Discussion

The reason for evaluating CPF as a human milk liquid fortification product in situations where there was reluctance by the clinical team to use powder HMF was the potential advantage of a liquid product in mixing and lower osmolality without compromising growth outcomes. In our unit, the clinical team (surgical and medical) did not wish to start (or restart) powder HMF in a certain group of neonates due to our previous experience of intestinal obstruction in high-risk babies on powder HMF [24]. Since Health Canada did not approve any other fortifier products apart from powder HMF and CPF for use in preterm infants, we felt the need to test this alternative option for high-risk babies. Our intent was to report the experience of using this product as a liquid HMF in this cohort. Conducting a prospective randomized trial with CPF was not considered appropriate, as CPF displaces a significant volume of human milk. To our knowledge, this is the first report describing tolerance and outcomes of CPF as a liquid HMF in babies considered at increased risk of feed intolerance. Addition of CPF as a liquid fortifier displaces 40% of human milk volume, which is a disadvantage; however, the more concentrated commercial liquid bovine HMFs used in other centers [40] were not available in Canada at the time of the study. Addition of multiple products can increase the osmolality of the milk and further contribute to issues with tolerance [19]. A previous clinical trial comparing CPF to powder HMF did not show a difference in weight gain or tolerance between the two groups. They compared two nutritional products from the same manufacturer [27]. However, their study group comprised all very low birth weight (VLBW) babies compared to our cohort of babies at increased risk of feed intolerance. Feeding intolerance is a widely described outcome variable in studies involving preterm enteral nutrition [41]. The overall incidence of feed intolerance in our group was 14%. However, out of the 4 babies who showed intolerance, CPF was restarted in one baby, as the medical team did not attribute the intolerance to CPF. Moreover, one baby was receiving additional protein additive at the time intolerance was noted. Kim et al. [36] conducted a randomized controlled trial comparing powder HMF versus liquid hydrolysed fortifier among preterm babies <33 weeks and birth weights ranging between 700–1500 g. The incidence of babies with feed intolerance in their cohort was 23% in the liquid fortifier group and 19% in the powder HMF group. A retrospective study by Thoene and colleagues [42] compared outcomes of babies on non-acidified liquid HMF, acidified liquid HMF, and powder HMF. Their cohort comprised bigger babies with fewer medical morbidities. Although they did not report feeding intolerance as an outcome, the incidence of NEC in the acidified liquid HMF group was 13%, compared to nil in the powder HMF group. Mukhopadhyay et al. [43] enrolled babies <1500 g and <34 weeks in a randomized trial, comparing powder HMF fortification and exclusive human milk feeding with vitamin and mineral supplementation. They found that the incidence of feed intolerance (vomiting, abdominal distension, and increased aspirates) was 21% in the powder HMF group and 29% in the control group. These studies report similar or higher incidence of feed intolerance than our study population, which suggests that CPF is well tolerated, especially since our population consisted of babies that were at a higher baseline-risk for feed intolerance.

There is no international consensus regarding what constitutes the ideal growth pattern for premature babies, especially those born very preterm [44]. The median GV of our study population throughout the NICU stay was 13.4 g/kg/day. However, once feeds were fortified with CPF to 24 kal/oz, the median GV improved to 15.9 g/kg/day. In a prospective randomized trial comparing ultra-concentrated liquid HMF with powder HMF, Moya and colleagues demonstrated a mean weight gain of 15.8 g/kg/day in the liquid HMF group. They included infants ≤1250 g and the liquid HMF used in that study provided around 20% more protein compared to powder HMF [45]. The previous clinical trial using concentrated preterm formula demonstrated that VLBW babies on CPF had a mean weight gain of 18.3 g/kg/day compared to 16.9 g/kg/day in the powder HMF group [27]. In the retrospective study by Thoene et al., the growth rates reported were 15.4 g/kg/day in the powder HMF group, 10.6 g/kg/day in the acidified liquid HMF group, and 14 g/kg/day in the non-acidified liquid HMF group, respectively [42]. There are limited data on the growth outcomes of babies at high risk of feed intolerance. A multicenter retrospective study by Hintz et al. looked at growth outcomes of babies post NEC (surgical and medical NEC). They reported a weight gain of 13 g/kg/day [46]. Although only one-fourth of babies in our group had intestinal surgery, their median growth velocity post CPF fortification was 18.9 g/kg/day (*n* = 7). Mukhopadhyay et al. reported that the weight gain in babies on fortified human milk was 15 g/kg/day versus 12.9 g/kg/day in the unfortified milk group [43]. In another trial involving very low birth weight (VLBW) infants, Nicoll et al. achieved a mean weight gain of 15.1 g/kg/day in the fortified group compared to 13.2 g/kg/day in the unfortified group [47]. Ehrenkranz et al. have reported that with improved weight gain in hospitalized preterm infants, the incidence of neurodevelopmental impairment and need for rehospitalisation decreased significantly. A rate of weight gain >18 g/kg/day and an HC growth rate of >0.9 cm/week were associated with better neurodevelopmental and growth outcomes [48]. Although the observed growth in our study population was below the optimal target, the growth velocity markedly improved after addition of CPF. Growth results in our cohort need to be interpreted with caution, as some babies received additional nutritional components (protein or lipid supplements) while on CPF, as decided by the clinical team, since the primary purpose of this study was to assess for tolerance and safety. Those babies also demonstrated similar growth and were not adversely affected by the supplements. Thus, the nutritional adequacy and tolerance of human milk fortified with CPF needs to be monitored closely by the clinical team. Depending on the feed volume, type of milk (donor or mother’s milk), and growth pattern, individualized supplementation may be required after assessment by the team.

Acidification of milk has been shown to cause changes in nutritional and cellular composition [49]. Thoene et al. reported a higher incidence of metabolic acidosis and slower growth in infants receiving an acidified liquid HMF compared to those receiving powder HMF [50]. In our study, none of the babies on CPF developed metabolic acidosis or significant biochemical abnormalities. One of the reasons for not having metabolic acidosis in our cohort could be the late addition of CPF fortifier to feeds, as the acid buffering mechanisms are poor in the first few weeks of life in a preterm infant [51].

Our study has a few limitations, including the lack of a control group, small sample size, and some missing weekly data on length and head circumference. The strengths of our study include its prospective design and inclusion of preterm babies with previous history of feed or HMF intolerance and babies with surgical bowel. While the issue of displacement of volume of human milk due to a liquid fortifier could be considered a disadvantage, depending on whether sufficient mother’s milk is available, this can be an advantage in selective cases.

## 5. Conclusions

The use of CPF formula as a liquid fortifier in preterm babies at increased risk for feed intolerance appears to be well tolerated and facilitates growth. In a center with limited liquid human milk fortification options, CPF provided an additional/useful alternative for this challenging subgroup of preterm babies. However, we recommend using CPF with close monitoring of growth and biochemical parameters to ensure nutritional adequacy.

## Figures and Tables

**Table 1:** Comparison of powder human milk fortifier (HMF) and concentrated preterm formula (CPF).

24 kcal/oz Fortified Preterm Human Milk *		
**Per 100 mL**	**Powder HMF**	**CPF**
**Dilution/Mixing**	4 packets to 100 mL HM	40 mL CPF with 60 mL HM
**Calories**	81	81
**Protein**	2.6	2.1
**Iron**	1.48	0.74
**Ca**	119	90.6
**P**	59.3	46
**Vitamin D**	154	63
**Osmolality**	325	304

* Preterm human milk nutrient values adapted from published literature [28,29,30].

**Table 2:** Patient characteristics.

*n* = 29	Value
Gestation age, week + day; median (IQR)	26 + 3 (24 + 6–28 + 2)
Birth weight, g; median (IQR)	833 (635–1050)
Male sex; *n* (%)	20 (68.9%)
C section; *n* (%)	20 (68.9%)
Enteral feeds start day; median (IQR)	2 (2–5)
Day of life when full feeds achieved; median (IQR)	30 (16–53)
TPN days; median (IQR)	29 (17–61)
CPF start day; median (IQR)	47 (31–60)
Number of days CPF received; median (IQR)	28 (13–39)
Retinopathy of Prematurity (ROP) needing treatment *; *n* (%)	6 (20.6%)
Chronic Lung Disease (CLD)^;^ *n* (%)	18 (62.1%)
Late onset sepsis (blood) ^$^	10 (34.5%)
Metabolic bone disease	13 (44.8%)
Discharge/Transfer gestation week; median (IQR)	39 (36–44)
Discharge/Transfer weight g; median (IQR)	2795 (2300–3907)

* ROP requiring laser therapy, Avastin, or surgery ^$^ Blood culture positive cases.

**Table 3:** Characteristics of babies developing any feed intolerance.

Baby	GA *	Birth Weight (g)	Day of Start of CPF	Days on CPF When Intolerance Noted	Abdominal Distension	Emesis	Change in Stool/Stoma Output	Clinical/Culture Positive Sepsis	If Restarted on CPF
A	27 + 1	1000	58	2	No	Yes	No	Clinical	No
B	24 + 0	840	47	10	No	No	Yes	No	No
C	28 + 0	574	40	21	Yes	No	No	Clinical	Yes
D	29 + 6	1135	29	28	Yes	No	No	Culture	No

* Gestational Age, week + day.

**Table 4:** Growth and laboratory data on CPF **(***n* = 23, unless specified otherwise).

Characteristic	Value
Total observation days on CPF median (IQR)	34 (24–49)
Days on CPF 24 kcal/oz median (IQR)	27 (12.5–35.5)
Growth velocity prior to CPF (g/kg/day) median (IQR)	12.53 (11.0–15.4)
Growth velocity on CPF (g/kg/day) median (IQR)	15.87 (11.7–19.0)
Weight at start of CPF (grams) median (IQR)	1500 (1254–1746)
Weight at end of CPF (grams) median (IQR)	2128 (2500–2778)
Weight gain on CPF (g/day) median (IQR)	31.4 (22.9–36.2)
Head growth prior to CPF (*n* = 13) cm/week median (IQR)	0.75 (0.53–0.76)
Head growth on CPF (*n* = 13) cm/week median (IQR)	0.79 (0.69–0.86)
Length growth prior to CPF (*n* = 12) cm/week median (IQR)	0.88 (0.84–0.93)
Length growth on CPF (*n* = 12) cm/week median (IQR)	0.77 (0.67–1.08)
Maximum BUN (*n* = 27) mmol/L median (IQR)	2.4 (1.25–4)
Maximum pre-albumin (*n* = 27) mg/L median (IQR)	103 (73.5–119.5)
